# The contribution of morbidity and unemployment for the reduced labor market participation of individuals with neurofibromatosis 1 in Finland

**DOI:** 10.1038/s41431-023-01426-5

**Published:** 2023-07-18

**Authors:** Roope A. Kallionpää, Edvard Johansson, Petri Böckerman, Juha Peltonen, Sirkku Peltonen

**Affiliations:** 1 Cancer Research Unit and FICAN West Cancer Centre, Institute of Biomedicine, University of Turku, Turku, Finland; 2 Faculty of Social Sciences, Business, and Economics, Åbo Akademi University, Turku, Finland; 3 Jyväskylä University School of Business and Economics, Jyväskylä, Finland; 4 Labour Institute for Economic Research LABORE, Helsinki, Finland; 5 IZA Institute of Labor Economics, Bonn, Germany; 6 Department of Dermatology and Venereology, University of Turku, Turku, Finland; 7 Department of Dermatology, Turku University Hospital, Turku, Finland; 8 Department of Dermatology and Venereology, Institute of Clinical Sciences, Sahlgrenska Academy, University of Gothenburg, Gothenburg, Sweden; 9 Department of Dermatology and Venereology, Region Västra Götaland, Sahlgrenska University Hospital, Gothenburg, Sweden; 10 Department of Dermatology and Allergology, University of Helsinki, Helsinki, Finland; 11 Skin and Allergy Hospital, Helsinki University Hospital, Helsinki, Finland

**Keywords:** Health care economics, Clinical genetics, Cancer epidemiology, Cancer epidemiology, Epidemiology

## Abstract

Neurofibromatosis 1 (NF1) is a multisystem disorder associated with, for example, a high risk for cancer, a variety of behavioral and cognitive deficits, low educational attainment and decreased income. We now examined the labor market participation of individuals with NF1. We analyzed the numbers of days of work, unemployment, and sickness allowance among 742 Finnish individuals with NF1 aged 20–59 years using nationwide register data from Statistics Finland and the Social Insurance Institution of Finland. The individuals with NF1 were compared with a control cohort of 8716 individuals matched with age, sex, and the area of residence. Individuals with NF1 had a significantly lower number of working days per year than the controls (rate ratio [RR] 0.93, 95% CI 0.91–0.95). Unemployment (RR 1.79, 95% CI 1.58–2.02), and sickness absence (RR 1.44, 95% CI 1.25–1.67) were more frequent in the NF1 than in the control group. The causes of sickness allowances were highly concordant with the previously reported morbidity profile of NF1 including neoplasms, cardiovascular disease, mental and behavioral diseases, and neurological diseases. In conclusion, NF1 significantly interferes with labor market participation *via* both unemployment and morbidity. Unemployment seems to cause more days of not working than sickness absence.

## Introduction

Neurofibromatosis 1 (NF1; OMIM 162200) is a monogenic disorder with an average prevalence of 1/3000 to 1/2000 [1, 2]. The NF1 syndrome is caused by pathogenic variants of the *NF1* gene in chromosome 17 [3, 4]. The diagnosis of NF1 is based on clinical criteria, with or without genetic analysis [5, 6]. The syndrome is characterized by cutaneous pigmentary findings, such as café-au-lait macules and skinfold freckling, and the benign hallmark tumors, neurofibromas. Cutaneous neurofibromas never undergo malignant degeneration, yet their number may exceed thousands in some individuals [7–9], and they may cause a significant psychological burden [10–12]. Plexiform neurofibromas are considered congenital, and are often located in visceral tissues or extremities. Plexiform neurofibromas may cause disfigurement and functional deficits, and they may become malignant [13–15].

NF1 is a cancer predisposition syndrome associated with approximately 60% lifetime risk for cancer [16]. The cancer risk in NF1 is significant throughout the lifetime [16] and involves, for example, tumors of the central and peripheral nervous system, breast cancer, and gastrointestinal tumors [16–19]. In addition to cancers, individuals with NF1 have increased risks for, for example, cardiovascular disease, osteoporosis, scoliosis, dementia, and chronic pain [20–24]. NF1 causes excess mortality throughout the lifetime and is associated with a shortened life expectancy [1, 2, 25, 26]. Consequently, the prevalence of NF1 declines in older age groups [2].

Cognitive and behavioral disorders such as attention-deficit-hyperactivity disorder, and deficits in executive functioning, difficulties of speech, reduced IQ, and impairments in general cognition, visuospatial processing and motor abilities are common among individuals with NF1 [27–35]. Individuals with NF1 show notably decreased educational attainment, and a tendency to obtain vocational instead of academic education [35, 36]. The cancer-related morbidity, and developmental and cognitive disorders are major contributors to the lower educational attainment of individuals with NF1 [35]. Parental NF1 may also reduce the educational attainment [35, 37]. We recently reported that NF1 causes lower income and increased use of social income transfers in Finland [38]. The lower income in the NF1 than in the control group was associated with individuals with NF1 working a lower number of months per year than controls, and increased numbers of hospital visits and sick days among individuals with NF1 [38]. A recent study found that perceived barriers to employment impact the quality of life, anxiety, and depression of individuals with NF1 more than those of matched controls [39], which highlights the need for more information on employment in NF1.

The morbidity associated with NF1, and the lower educational level, disfigurement, and cognitive difficulties that can interfere with employment, are likely to contribute to the decreased economic well-being and reduced labor market participation of individuals with NF1. In the present study, we aim at dissecting the roles of unemployment, sickness absence, and disability in the labor market participation of individuals with NF1. Moreover, using register-based information, we analyze the co-morbidities underlying the sickness and disability allowances observed among individuals with NF1. The results pave the way for interventions intended to support and improve the ability of individuals with NF1 to work and to find suitable employment.

## Materials and methods

The study was based on the previously described Finnish NF1 cohort [1]. The cohort has been collected by searching all hospital visits related to NF1 from the 5 university hospitals and 15 central hospitals of mainland Finland in 1987–2011. The medical records of the identified individuals were reviewed to confirm the fulfillment of the National Institutes of Health (NIH) diagnostic criteria for NF1 [1, 5]. Individuals with clearly segmental NF1 phenotype were excluded. For each individual with NF1, a maximum of ten control individuals matched with age, sex, and the area of residence at cohort entry were retrieved from the Finnish Population Register Centre. The first-degree relatives of individuals with NF1 were excluded from the control cohort.

The Finnish personal identity codes allow comprehensive linkage of national register data to each person over time. Individuals with NF1 and controls were followed up starting from their entry into the cohort, the start of the study period, or the date of reaching the lower age limit of each analysis, whichever occurred last. The follow-up ended at death, emigration, the date of reaching the upper age limit, or the end of the study period.

### The numbers of days of working, sickness allowance, and unemployment

The numbers of working days, reimbursed days of sickness absence and days of unemployment were examined among individuals aged 20–59 years. The analysis was focused on individuals ≥20 years because the vast majority of Finnish adolescents aged <20 years are students and would have been excluded from the analysis. The analysis was limited to those <60 years of age because age-related retirement in Finland mostly occurs at ages ≥60 years. The data on labor market status and unemployment were obtained from Statistics Finland for the study period of 2005–2015. Unemployment is defined based on official registration as a job seeker, which is mandatory to obtain the public unemployment benefits. In case of sickness, the employees are entitled to normal salary for the first nine days, after which the Social Insurance Institution of Finland provides sickness allowance as a compensation for incapacity to work. Thus, the data do not include short sickness absence spells. Students, conscripts, and pensioners were excluded from the analyses.

Poisson regression was used to compare the yearly numbers of working days, reimbursed days of sickness absence and days of unemployment between the NF1 and control groups. The models were adjusted for age and sex. In additional analyses, the models were also adjusted for educational level, or for educational level and history of cancer within the three years preceding each year of interest. The educational level of each individual was obtained from the register maintained by Statistics Finland and coded according to the International Standard Classification of Education [35]. The history of cancer was based on data from the Finnish Cancer Registry. Standard errors were clustered within the strata of each individual with NF1 and the matched controls to account for the dependency stemming from the matching of the controls. The analyses were performed using the Stata software version 17.

### The causes of sickness allowances

To further analyze the causes of sickness allowances, a diagnosis-specific analysis was performed among individuals aged 20–59 years based on the data from the Social Insurance Institution of Finland. Payments of sickness allowance require a medical certificate, from where the diagnosis codes recorded in the register are obtained. The analysis encompassed the years since the introduction of the International Classification of Diseases, 10^th^ edition (ICD-10) in Finland, that is, over the period 1996–2014. Individuals with a disability pension or rehabilitation subsidy granted prior to 1996 were excluded, and the follow-up ended at the beginning of a newly granted disability pension or rehabilitation subsidy.

The analysis was stratified by the chapters of the ICD-10 classification. The categories associated with sickness allowance in less than three individuals with NF1 or less than three controls were excluded. The numbers of reimbursed days of sickness were analyzed using generalized linear mixed effects regression with Poisson distribution and a random intercept for the clusters of each individual with NF1 and the matched controls. The follow-up time of each individual was included as a model offset with a log-link. The analysis was performed with the R software version 4.0.0 and package lmerTest version 3.1-2.

### The risk and causes of disability-related pension

Sickness allowance can only be paid for a limited time. Individuals with a prolonged incapacity to work because of sickness may qualify for a fixed-term disability pension, also termed rehabilitation subsidy, and intended for returning to work in, for example, a different profession. A permanent disability pension can be granted for those who cannot regain their ability to work. These two disability-related pensions are administered and registered by the Social Insurance Institution of Finland. For each pension, up to three causative diagnosis codes are registered.

The present analysis encompassed the years 1996–2014 and individuals aged 18–59 years. The lower age limit of the analysis was set at 18 years to cover those individuals who are granted disability pension as soon as they come of age. The follow-up ended at the first disability-related pension, that is, those returning to work after a fixed-term disability pension were not allowed to re-enter the analysis. The time-to-pension was analyzed with age as the time scale. The Kaplan–Meier estimate of the cumulative risk for any disability-related pension, that is, permanent disability pensions and rehabilitation subsidies combined, was computed while accounting for the competing risk of death and allowing delayed entry. In another analysis, the competing risks of disability pension, rehabilitation subsidy and death were included.

Cox proportional hazards models were used to estimate the hazard ratios (HRs) with their 95% confidence intervals (CIs) for disability pension, rehabilitation subsidy, or either of these two for each of the ICD-10 chapters. All three registered diagnosis codes were considered in the analyses, and one pension could therefore be counted in multiple diagnosis groups. The diagnosis categories associated with a disability-related pension in less than three individuals with NF1 or less than three controls were excluded from the analysis. The matching of individuals with NF1 and controls was accounted for by including a frailty term in the models. This implies that the estimated models account for unobserved heterogeneity by including random effects. The proportional hazards assumption was assessed visually and using scaled Schoenfeld residuals. The analyses were performed using the R software version 4.0.0 and package survival version 3.1–12.

## Results

### The days of working, sickness allowance and unemployment

A total of 742 individuals with NF1 and 8716 controls aged 20–59 years contributed 5224 and 66,458 person-years of follow-up time in 2005–2015, respectively (Supplementary Table). The average follow-up time was 7.0 years per individual with NF1 and 7.6 years per control individual.

**Fig. 1:**
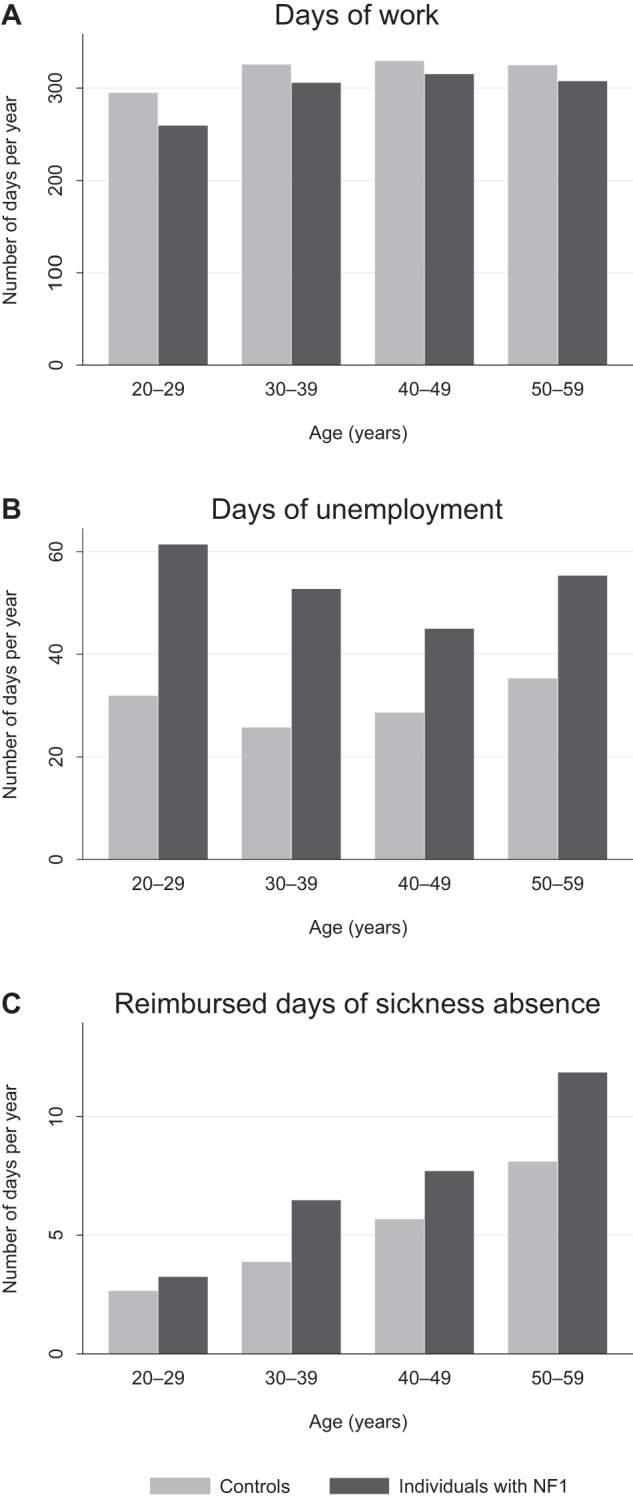
The numbers of days of work, unemployment and sickness absence among individuals with neurofibromatosis 1 (NF1) and matched controls. The average numbers of days of work (**A**), days of unemployment (**B**), and reimbursed days of sickness absence (**C**) per year by age group. The figure represents raw observed values.

Individuals with NF1 had a lower number of working days than the controls irrespective of the age group (Fig. 1A). The number of working days per year was statistically significantly lower among individuals with NF1 than among controls (rate ratio [RR] 0.93; Table 1), and the effect persisted after adjustment with educational level, or educational level and history of cancer in addition to age and sex (Table 1).

**Table 1:** The rate ratios (RRs) and their 95% confidence intervals (CI) showing the comparison of the numbers of days of working, unemployment and sickness allowance among 742 individuals with NF1 versus 8716 control individuals followed up at ages 20–59 in 2005–2015.

	RR (95% CI) adjusted for age and sex	RR (95% CI) adjusted for age, sex and education	RR (95% CI) adjusted for age, sex, education and 3-year history of cancer
Days of working	0.93 (0.91–0.95)	0.94 (0.92–0.96)	0.94 (0.92–0.96)
Days of unemployment	1.79 (1.58–2.02)	1.58 (1.40–1.79)	1.59 (1.40–1.80)
Reimbursed days of sickness	1.44 (1.25–1.67)	1.30 (1.12–1.50)	1.17 (1.01–1.34)

Individuals with NF1 showed markedly higher rates of unemployment than controls irrespective of age (Fig. 1B), and the difference was significant after adjustment with age and sex (RR 1.79; Table 1). As expected, the number of reimbursed days of sickness absence correlated with older age, yet individuals with NF1 had more reimbursed days of sickness absence than controls in all age groups (Fig. 1C) with an overall RR of 1.44 (Table 1). After adjusting for educational level, the RRs for days of unemployment and reimbursed days of sickness absence were 1.58 and 1.30, respectively (Table 1), indicating that the effect of NF1 was partially mediated by the lower educational attainment among individuals with NF1. The effect of NF1 on the number of days of unemployment remained essentially unchanged after adjustment also for the history of cancer within the past three years. However, the history of a recent cancer explained a large proportion of the reimbursed days of sickness absence with an adjusted estimate of 1.17 for the effect of NF1 (Table 1).

### Diagnoses associated with sickness allowances

Among the 850 individuals with NF1, and 9423 controls aged 20–59 years in 1996–2014 (Supplementary Table), 473 (56%) individuals with NF1 and 4376 (46%) controls had at least one period of sickness allowance within an average follow-up time of 9.8 years (SD 6.2) per individual with NF1 and 11.0 years (SD 6.1) per control. Several groups of ICD-10 diagnoses were associated with a higher number of reimbursed days of sickness absence among individuals with NF1 than among controls, yet some ICD-10 chapters also showed an inverse association (Table 2).

**Table 2:** The numbers of reimbursed days of sickness absence among individuals with neurofibromatosis 1 (NF1) and matched controls.

ICD-10 chapter (ICD-10 codes)	Number of individuals with sickness allowance in the NF1 cohort of 850 individuals, *n* (%)	Number of individuals with sickness allowance in the control cohort of 9423 individuals, *n* (%)	Number of reimbursed days of sickness out of the total of 66,065 days in the NF1 cohort, *n* (%)	Number of reimbursed days of sickness out of the total of 458,436 days in the control cohort, *n* (%)	The counts of reimbursed days of sickness in NF1 vs. control cohort, RR (95% CI)
Certain infectious and parasitic diseases (A00-B99)	9 (1.1)	109 (1.2)	60 (0.1)	2182 (0.5)	0.34 (0.26–0.44)
Neoplasms (C00-D48)	99 (11.6)	284 (3.0)	13,336 (20.2)	28,097 (6.1)	7.41 (7.25–7.57)
Diseases of the blood and blood-forming organs and certain disorders involving the immune mechanism (D50-D89)	4 (0.5)	12 (0.1)	236 (0.4)	378 (0.1)	11.45 (9.56–13.7)
Endocrine, nutritional and metabolic diseases (E00-E90)	14 (1.6)	70 (0.7)	652 (1.0)	3969 (0.9)	1.91 (1.75–2.07)
Mental and behavioral disorders (F00-F99)	112 (13.2)	1089 (11.6)	11,300 (17.1)	110,391 (24.1)	1.29 (1.26–1.31)
Diseases of the nervous system (G00-G99)	35 (4.1)	297 (3.2)	3205 (4.9)	19,051 (4.2)	2.08 (2.00–2.16)
Diseases of the eye and adnexa, ear and the mastoid process (H00-H95)	14 (1.6)	119 (1.3)	210 (0.3)	3892 (0.8)	0.58 (0.50–0.67)
Diseases of the circulatory system (I00-I99)	31 (3.6)	302 (3.2)	3311 (5.0)	19,254 (4.2)	2.28 (2.19–2.37)
Diseases of the respiratory system (J00-J99)	30 (3.5)	426 (4.5)	569 (0.9)	8175 (1.8)	0.92 (0.85–1.00)
Diseases of the digestive system (K00-K93)	30 (3.5)	379 (4.0)	890 (1.3)	9624 (2.1)	1.13 (1.05–1.21)
Diseases of the skin and subcutaneous tissue (L00-L99)	8 (0.9)	100 (1.1)	136 (0.2)	3318 (0.7)	0.53 (0.44–0.63)
Diseases of the musculoskeletal system and connective tissue (M00-M99)	134 (15.8)	1575 (16.7)	11,177 (16.9)	140,979 (30.8)	1.04 (1.02–1.06)
Diseases of the genitourinary system (N00-N99)	16 (1.9)	201 (2.1)	235 (0.4)	4467 (1.0)	0.63 (0.55–0.72)
Pregnancy, childbirth and the puerperium (O00-O99)	24 (2.8)	292 (3.1)	960 (1.5)	8940 (2.0)	1.28 (1.20–1.37)
Congenital malformations, deformations and chromosomal abnormalities (Q00-Q99)	124 (14.6)	18 (0.2)	11,323 (17.1)	1024 (0.2)	462 (425–504)
Symptoms, signs and abnormal clinical and laboratory findings, not elsewhere classified (R00-R99)	20 (2.4)	187 (2.0)	1065 (1.6)	5080 (1.1)	2.84 (2.65–3.04)
Injury, poisoning and certain other consequences of external causes (S00-T98)	72 (8.5)	1067 (11.3)	3500 (5.3)	53,735 (11.7)	0.79 (0.77–0.82)

*CI* confidence interval, *ICD-10* International Classification of Diseases, 10^th^ edition, *RR* rate ratio.

Neoplasms (ICD-10 C00-D48) were a significantly more frequent cause of sickness allowance in the NF1 group than in the control group (RR 7.41, 95% CI 7.25–7.57), contributing 20% of the reimbursed days of sickness absence among individuals with NF1 and 6% among the controls (Table 2). Mental and behavioral diseases (ICD-10 F00-F99) and diseases of the musculoskeletal system and connective tissue (ICD-10 M00-M99) were other major causes of sickness allowance (Table 2). Out of the total number of reimbursed days of sickness, the proportions of mental and behavioral diseases, and diseases of the musculoskeletal system and connective tissue were smaller among individuals with NF1 than among controls. However, these diagnoses still caused more days of sickness in the NF1 than in the control group (RR 1.29, 95% CI 1.26–1.31 and RR 1.04, 95% CI 1.02–1.06, respectively). As expected, congenital malformations, deformations, and chromosomal abnormalities (ICD-10 Q00-Q99) were highly overrepresented among individuals with NF1 (RR 462, 95% CI 425–504). However, this finding merely reflects the registration of the NF1 itself (ICD-10 Q85) as the cause of the sickness allowance, which was the case in 96% of the sickness allowances due to congenital malformations, deformations, and chromosomal abnormalities among individuals with NF1.

### Diagnoses contributing to disability-related pensions

**Fig. 2:**
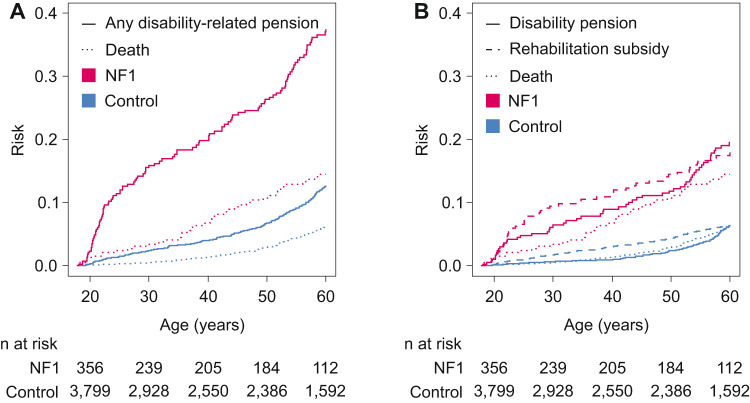
The cumulative risk of pension related to incapacity to work among individuals with neurofibromatosis 1 (NF1) and controls. **A** The risk for any disability-related pension including both permanent and fixed-term pensions with death as a competing risk. **B** The competing risks for permanent disability pension, rehabilitation subsidy (fixed-term disability pension), and death. The numbers of individuals at risk at different ages are shown below the figure.

Among the 924 individuals with NF1 and 10,126 controls followed up over age 18–59 years in 1996–2014 (Supplementary Table), NF1 was associated with increased risks of any disability-related pension, a permanent disability pension, and a fixed-term disability pension, also termed rehabilitation subsidy (Fig. 2). All the ICD-10 chapters associated with disability-related pensions (Table 3) were also highlighted in the analysis of the sickness allowances (Table 2). The NF1 itself was the sole cause of the disability-related pension in 10 (8.9%) individuals with NF1 and disability-related pension. The cumulative risk for any disability-related pension was substantially higher among individuals with NF1 than among controls irrespective of age (Fig. 2; Table 4). Individuals with NF1 had roughly equal cumulative risks of permanent disability pension and fixed-term rehabilitation subsidy by the age of 60 years. In the NF1 group, 58% of the individuals with a fixed-term disability pension obtained a permanent disability pension later during the follow-up, while the proportion was 50% among the controls (*P* = 0.284). The corresponding proportions in the NF1 and control groups were 53% and 27% among individuals younger than 30 years (*P* = 0.022), and 67% and 76% among individuals aged 50–59 years (*P* = 0.681), respectively.

**Table 3:** The risks of permanent disability pension, rehabilitation subsidy (fixed-term disability pension), and either of the two, i.e., any disability-related pension among 924 individuals with neurofibromatosis 1 (NF1) and 10,126 controls aged 18–59 years in 1996–2014.

Diagnosis (ICD-10)	All disability-related pensions			Permanent disability pension			Rehabilitation subsidy		
	*n*, NF1	*n*, Control	HR (95% CI)	*n*, NF1	*n*, Control	HR (95% CI)	*n*, NF1	*n*, Control	HR (95% CI)
All diagnoses combined	112	334	4.25 (3.43–5.27)^a^	57	159	4.88 (3.60–6.63)^a^	55	175	3.87 (2.86–5.24)^a^
Neoplasms (C00-D48)	29	27	13.8 (8.14–23.3)^a^	13	16	10.8 (5.15–22.4)^a^	16	11	17.8 (8.24–38.3)
Endocrine, nutritional and metabolic diseases (E00-E90)	3	14	2.75 (0.79–9.59)	–	–	–^b^	–	–	–^b^
Mental and behavioral disorders (F00-F99)	43	183	2.89 (2.08–4.04)^a^	20	79	3.22 (1.97–5.26)^a^	23	104	2.66 (1.69–4.18)
Diseases of the nervous system (G00-G99)	16	40	5.14 (2.88–9.19)	10	30	4.34 (2.12–8.89)	6	10	7.45 (2.71–20.5)
Diseases of the circulatory system (I00-I99)	8	30	3.55 (1.62–7.75)	4	21	2.61 (0.89–7.61)	4	9	5.55 (1.71–18.1)
Diseases of the musculoskeletal system and connective tissue (M00-M99)	16	85	2.58 (1.51–4.41)	9	41	3.17 (1.53–6.55)	7	44	2.07 (0.93–4.59)
Congenital malformations, deformations and chromosomal abnormalities (Q00-Q99)	60	4	180 (65.4–495)^a^	–	–	–^b^	–	–	–^b^

*CI* confidence interval, *HR* hazard ratio, *ICD-10* International Classification of Diseases, 10^th^ edition.

^a^The Cox proportional hazards model assumption regarding the proportionality of the hazards was not met.

^b^The estimates are not shown for analyses with <3 individuals in either group.

**Table 4:** The Kaplan–Meier estimates of the risks for permanent disability pension, rehabilitation subsidy (fixed-term disability pension), and either of the two, i.e., any disability-related pension among individuals with neurofibromatosis 1 (NF1) and controls.

	Risk of any disability-related pension, % (95% CI)^a^		Risk of permanent disability pension, % (95% CI)^b^		Risk of fixed-term disability pension (rehabilitation subsidy), % (95% CI)^b^	
	NF1	Control	NF1	Control	NF1	Control
By age 30	15.9 (12.4–20.3)	2.4 (1.9–3.0)	6.4 (4.3–9.6)	0.7 (0.4–1.0)	9.5 (6.8–13.2)	1.7 (1.4–2.2)
By age 40	19.9 (15.9–24.8)	4.0 (3.4–4.8)	9.0 (6.3–12.8)	0.9 (0.7–1.4)	10.9 (8.0–14.9)	3.1 (2.5–3.7)
By age 50	26.4 (21.8–31.8)	6.8 (5.9–7.8)	11.9 (8.7–16.1)	2.4 (1.9–3.1)	14.5 (11.0–19.0)	4.4 (3.7–5.2)
By age 60	37.5 (32.2–43.5)	12.7 (11.5–14.1)	19.5 (15.4–24.7)	6.4 (5.5–7.5)	17.9 (14.1–22.9)	6.2 (5.4–7.2)

The numbers of individuals at risk at each time point are shown under Fig. 2.

*CI* confidence interval.

^a^The estimates were computed allowing for the competing risk of death.

^b^Permanent disability pension, fixed-term disability pension, and death were included as competing risks.

New disability-related pensions began at all ages, yet the youngest and oldest individuals with NF1 displayed a particularly steep increase in the risk for a disability-related pension (Fig. 2A). Among the 22 individuals with NF1 who were granted a permanent disability pension at an age younger than 30 years, 11 (50%) had mental disability (ICD-10 F70-F79) as a contributing diagnosis, while mental disability was mentioned in 13/23 (57%) of the controls. Cancer was recorded as a cause of a permanent disability pension in five (23%) young individuals with NF1 and in none of the controls. In addition, the NF1 itself (ICD-10 Q85) was recorded in association with permanent disability pension in 16 (73%) of the individuals with NF1. Among the 32 individuals with NF1 and 60 controls with a fixed-term disability pension starting at an age <30 years, cancer was mentioned in 8 (25%) individuals with NF1 and 3 (5%) controls. Mood disorders (ICD-10 F30-F39) were recorded as a contributor of a fixed-term disability pension in 8 (25%) individuals with NF1 and 26 (43%) controls at ages <30 years.

Twenty and 90 permanent disability pensions began in the NF1 and control groups, respectively, at ages 50–59 years. Major contributing diagnoses included diseases of the musculoskeletal system and connective tissue (ICD-10 M00-M99; 7 (35%) individuals with NF1 and 33 (37%) controls), mental and behavioral disorders (F00-F99; 5 (25%) and 36 (40%)), and diseases of the nervous system (G00-G99; 5 (25%) and 15 (17%)). The NF1 itself was recorded as a cause of a permanent disability pension in 10 (50%) individuals with NF1. The same diagnoses also contributed to the fixed-term disability pensions among those aged 50–59 years.

The distinct age-profiles of NF1-associated cancer risk [16], and the cognitive and behavioral problems associated with NF1 [27–35] are well known. As a result, the proportional hazards assumption of the Cox model was not fulfilled in the analyses of pensions related to neoplasms (ICD-10 C00-D48), mental and behavioral disorders (ICD-10 F00-F99) and congenital malformations, deformations, and chromosomal abnormalities (ICD-10 Q00-Q99), since the difference in the hazards was larger among younger individuals.

## Discussion

The present results demonstrate that the average number of working days per year is lower among individuals with NF1 than among their matched controls in Finland. The main factors underlying this observation are a higher rate of unemployment and increased morbidity associated with NF1, causing sickness absence and early retirement. The working careers of individuals with NF1 seem to be shorter because of relatively high unemployment especially in the young age groups (Fig. 1) and a notable risk for early retirement in all age groups (Fig. 2). While these findings may not be surprising given the serious health problems faced by individuals with NF1, the relative contributions of unemployment and morbidity have not been previously described in NF1. Unemployment, sickness absence and early retirement because of incapacity to work clearly contribute to the previously described poor economic well-being of individuals with NF1. However, it is notable that the variables associated with unemployment and morbidity did not entirely explain the lower income, and the higher need for social income transfers in our previous analysis [38].

The diagnoses contributing to sickness allowances and disability-related pensions were only analyzed at the chapter-level of ICD-10. Moreover, the diagnoses recorded in association with these benefits may not be completely accurate nor comprehensive and they necessarily provide a simplistic representation of the complications faced by an individual. Nevertheless, the diagnostic codes demonstrate a morbidity profile highly concordant with the previous knowledge. As expected, the marked cancer predisposition associated with NF1 [16–19] translated into significant excesses of reimbursed days of sickness absence and disability-related retirement. Cancers also partly but not completely explained the high overall number of reimbursed days of sickness absence in the NF1 group. Similarly, cognitive disorders and mental and behavioral diseases [27–35], diseases of the nervous [23, 24, 40] and circulatory systems [1, 23, 25], certain complications related to pregnancy [41, 42], and skeletal disorders [20, 21] are known to be more common in NF1 than in the general population, and the present results demonstrated excess days of sickness absence related to these conditions. The NF1 itself was frequently recorded as the cause of the sickness allowance or disability-related pension, which may reflect multi-system, ill-defined or diverse symptoms, such as fatigue, cognitive difficulties or chronic pain that is known to be frequent among individuals with NF1 [22].

Notably, the rates of reimbursed days of sickness absence associated with infectious diseases, and injuries, poisonings and other external causes were lower among individuals with NF1 than among controls (Table 2). The underlying reasons for this observation are unclear. An unemployed person is unlikely to obtain sickness allowance for a mild disease, such as a short-term respiratory infection, since such diagnoses are made overwhelmingly in occupational health care to which unemployed persons are not entitled, and the official diagnosis has no effect on the person’s daily activities. The lower rate of these diagnoses may therefore be an artifact resulting from the high rate of unemployment in the NF1 group. Clearly, the high rate of unemployment observed in the present study may also translate into a lower risk of catching infections and being injured at work.

The risk of a disability-related pension steadily increased among individuals with NF1 throughout the working age, yet particularly steep increases were seen among the youngest and oldest individuals. We hypothesize that some individuals with NF1 may undergo special education but be incapable of work after finishing their schooling. This hypothesis is supported by the high rates of mental disability and NF1 itself recorded as the causes of disability-related pensions among the youngest individuals. The steepening of the Kaplan–Meier curve estimating the risk for any disability-related pension in the NF1 group after 50 years of age (Fig. 2A) may reflect a variety of processes. The overall high morbidity associated with NF1 may cumulate over increasing age and thereby decrease the capacity to work. NF1 is associated with reduced cognitive functioning [29, 34], and the general age-related decline of cognitive performance [43] may reach a clinically detectable level before age-related retirement and cause premature incapacity to work more often in NF1 than in the general population.

Despite the extensive morbidity associated with NF1, the average number of days of unemployment was much higher than the number of reimbursed days of sickness absence among the Finnish individuals with NF1 (Fig. 1). Obviously, morbidity may also contribute to unemployment. For example, mental or cognitive disorders, attention deficits and autism spectrum traits, pain and disfigurement could be hypothesized to interfere with seeking and obtaining stable employment, and such factors are difficult to identify in register-based data due to under-reporting. For example, childhood externalizing and internalizing symptoms have been previously reported to be associated with decreased employment earnings in adulthood [44]. We detected no significant effect of a recent cancer on unemployment. Our analysis demonstrated that accounting for the educational level slightly attenuated the estimates, indicating that the high rate of unemployment among individuals with NF1 is partly due to their lower educational attainment. However, the difference to controls remained marked even after this adjustment. The increasing demands of modern-day work may particularly affect individuals with NF1 whose educational attainment is, on average, lower than in the general population [35, 36]. Unemployment was most pronounced in the youngest age groups of individuals with NF1, which suggests that young persons with NF1 need active support in their attempts to gain employment.

The present study is based on Finnish nationwide registers, which needs to be considered in the interpretation of the results. The data provide a comprehensive means for following up large cohorts. However, the data heavily rely on the Finnish processes related to the admissions and payments of sickness allowances, pensions, and unemployment benefits. As a result, caution is required when drawing conclusions on the consequences of NF1 in different countries and societies where, for example, the retirement age or the economic incentives of finding employment may be different than in Finland. The hospital-based ascertainment of the Finnish NF1 cohort may also have introduced selection bias, leading to artificially high morbidity within the cohort. While the NF1 diagnoses of all individuals included in the Finnish NF1 cohort have been confirmed, the patients display high variation in disease severity and manifestations, which is typical to NF1 [6, 45]. The results therefore represent an average NF1 population and are not necessarily applicable to any particular patient. Since unemployment may lead to not applying for sickness allowance even when entitled to it, and morbidity may contribute to unemployment, it is difficult to accurately estimate the relative contributions of these mechanisms.

It seems that both unemployment and morbidity are important contributors to the reduced labor market participation of individuals with NF1. In addition to unemployment and days of sickness absence, individuals with NF1 showed significantly increased rates of disability pension, indicating a permanent withdrawal from the labor market. In many cases, such as advanced cancers, working may not be possible. However, we hypothesize that effective identification and treatment of NF1-associated conditions like chronic pain may improve the affected individuals’ capacity to work. Multidisciplinary care for individuals with NF1 can help tackling the various aspects of NF1. It is important to identify the individuals with NF1 who are at a risk for unemployment or prolonged sickness that could be prevented.

## Conclusions

The results show that individuals with NF1 are significantly more often unemployed, on sickness absence, or receive disability-related pension than matched controls. Since unemployment is a major contributor to the decreased number of working days in the NF1 group, the results highlight the need for active support for obtaining employment especially in young adults with NF1. The morbidity profile contributing to sickness absence and disability-related pensions among individuals with NF1 is highly concordant with the previous knowledge on the diseases associated with NF1. Timely prevention, diagnosis and treatment of NF1-associated morbidity may improve the affected individuals’ capacity to work. NF1 demonstrates that a rare disease can significantly interfere with working and have wide-ranging effects on economic prospects.

### Supplementary information

Supplementary Table

## Data Availability

Data are available upon request for researchers though data access is restricted. Please contact the Finnish National Institute for Health and Welfare and Statistics Finland for permission. Data can be requested from the corresponding author Prof. Juha Peltonen: Institute of Biomedicine, University of Turku, Kiinamyllynkatu 10, FI-20520 Turku, Finland; juhpel@utu.fi.
