# Supplementary material for: The contribution of morbidity and unemployment for the reduced labor market participation of individuals with neurofibromatosis 1 in Finland
Source: Eur J Hum Genet. 2023 Jul 18;32(1):83–90. doi: 10.1038/s41431-023-01426-5 (PMC10772102; doi:10.1038/s41431-023-01426-5)
Supplement: Supplementary file 1 — Supplementary Table [file 41431_2023_1426_MOESM1_ESM.docx]

**Supplementary Table.** Basic demographics of the neurofibromatosis 1 (NF1) and control cohorts included in each analysis.

|  | **NF1** | **Control** |  |
| --- | --- | --- | --- |
| **Days of working, sickness allowance and unemployment: individuals aged 20–59 years, 2005–2015** | | | |
| Number of individuals | 742 | 8,716 |  |
| Age at start of follow-up, mean (SD) | 33.3 (12.2) | 33.8 (12.4) |  |
| Age at end of follow-up, mean (SD) | 39.7 (13.0) | 40.8 (13.1) |  |
| Year of birth, mean (SD) | 1973.8 (14.3) | 1973.0 (14.4) |  |
| Follow-up time (person-years), mean (SD) | 7.0 (3.7) | 7.6 (3.5) |  |
| Follow-up time (person-years), sum | 5,224 | 66,458 |  |
| **Sickness allowances: individuals aged 20–59 years, 1996–2014** | | |  |
| Number of individuals | 850 | 9,423 |  |
| Age at start of follow-up, mean (SD) | 30.0 (11.9) | 30.8 (12.2) |  |
| Age at end of follow-up, mean (SD) | 39.8 (13.6) | 41.7 (13.6) |  |
| Year of birth, mean (SD) | 1972.5 (15.2) | 1971.2 (15.5) |  |
| Follow-up time (person-years), mean (SD) | 9.8 (6.2) | 11.0 (6.1) |  |
| Follow-up time (person-years), sum | 8,355.7 | 103,477.8 |  |
| **Disability-related pensions: individuals aged 18–59 years, 1996–2014** | | |  |
| Number of individuals | 924 | 10,126 |  |
| Age at start of follow-up, mean (SD) | 28.3 (12.5) | 29.2 (12.8) |  |
| Age at end of follow-up, mean (SD) | 38.1 (14.3) | 40.2 (14.3) |  |
| Year of birth, mean (SD) | 1974.3 (15.8) | 1972.9 (16.2) |  |
| Follow-up time (person-years), mean (SD) | 9.8 (6.3) | 11 (6.2) |  |
| Follow-up time (person-years), sum | 9,069.8 | 111,428.3 |  |
